# 

**Published:** 1864-03

**Authors:** 


*Contents.*

ORIGINAL COMMUNICATIONS........................................ 33

1. Report on the Yellow Fever Epidemic at Wilmington, N. C., in the Autumn of 1862, by Surgeon W. T. Wragg. 2. Case of Diffused Traumatic Femoral Aneurism, reported by Surgeon J. G. Dudley, First Division, Winder Hospital. 3. Aneurism of Femoral Artery cured by Ligature, reported by Surgeon E. Burke Haywood, General Hospital, No. 7, Raleigh, N. C. 4. Report of Eruptive Fevers treated in General Hospitals, Department of Virginia, from October 1, 1862, to January 31st, 1864, consolidated by Surgeon Wm. A. Carrington, Medical Director. 5. On the Use of Phytolacca Decandra in Camp Itch, by Surgeon John H. Claiborne, in charge of Petersburg Hospitals. 6. Gun-shot Wound of Lung, Liver and Intestines, reported by Surgeon R. W. Jeffery, in charge of the Naval Hospital, Savannah. 7. Case of Congenital Defect of the Rectum, reported by B. W. Robinson, M. D., Fayetteville, N. C. 8. Excision of the Superior Third of Humerus, by Surgeon Otis Frederic Manson, in charge of General Hospital, No. 24, Richmond, Va.

CONFEDERATE STATES HOSPITAL REPORTS.............. 41

Eighteen Cases of Gun-shot Wounds of the Head, observed at the General Hospital, Charlottesville, Virginia.

EDITORIAL AND MISCELLANEOUS.............................. 43

Treatment of Aneurism by Compression. Association to Purchase Artificial Limbs for Maimed Soldiers.

TRANSACTIONS OF THE ASSOCIATION OF ARMY AND NAVY SURGEONS............................................ 44

Debate on Tetanus.

CHRONICLE OF MEDICAL SCIENCE.............................. 47

1. Remarks concerning the Hygiene of Military Hospitals, extracted from a paper read before the Imperial Academy of Medicine at Paris, by M. H. Baron Larrey; Paris, 1861; [translated from the French and abridged by Professor J. L. Cabell, University of Virginia, Surgeon P. A. C. S.]

